# Development and validation of college students’ tuberculosis knowledge, attitudes and practices questionnaire (CS-TBKAPQ)

**DOI:** 10.1186/s12889-017-4960-x

**Published:** 2017-12-12

**Authors:** Hualin Jiang, Shaoru Zhang, Yi Ding, Yuelu Li, Tianhua Zhang, Weiping Liu, Yahui Fan, Yan Li, Rongqiang Zhang, Xuexue Ma

**Affiliations:** 1 Health Science Center, Xi’an Jiaotong University, Xi’an city, China; 2 Department of Pharmacy, Xijing Hospital, Fourth Military Medical University, Xi’an city, China; 3 Shaanxi Provincial Institute for Tuberculosis Control and Prevention, Xi’an city, China; 4 Shaanxi University of Chinese Medicine, Xi’an city, China

**Keywords:** Tuberculosis, College students, Knowledge, Attitudes and practices, Reliability, Validity, Diagnostic accuracy

## Abstract

**Background:**

China faces many challenges in controlling tuberculosis (TB). One significant challenge is the control of college students’ TB. In particular, cross-sectional studies of college students’ knowledge, attitudes and practices (KAP) in regard to TB have attracted substantial attention. However, few measurement tools have been developed to aid processes related to expert consultation, pre-testing, reliability and validity testing. Our study developed the College Students’ TB Knowledge Attitudes and Practices Questionnaire (CS-TBKAPQ) following the scale development steps.

**Methods:**

The construction of the CS-TBKAPQ was based on the Theory of Knowledge, Attitude, Belief, and Practice (KABP or KAP). The item pool was compiled from literature reviews and individual interviews. The reliability validation was assessed by calculating Cronbach’s α coefficient, the split-half reliability coefficient, and the test-retest reliability coefficient. Construct validity was assessed using exploratory factor analysis (EFA) and confirmatory factor analysis (CFA). The diagnostic accuracy was evaluated using the World Health Organization Advocacy, Communication and Social Mobilization KAP Survey Questionnaire (WHO-TBKAPQ) as the reference standard.

**Results:**

A total of 31 questionnaire items were proposed. Cronbach’s α coefficient, the split-half reliability coefficient and the test-retest reliability coefficient were 0.86, 0.78 and 0.91. Four factors that explained 62.52% of the total variance were also identified in EFA and confirmed in CFA. The CFA model fit indices were *x* ^*2*^ */df* = 1.82 (*p* < 0.001), GFI = 0.925, AGFI = 0.900, RMR = 0.068, and RMSEA = 0.049. The CS-TBKAPQ was significantly correlated with the WHO-TBKAPQ and the Chinese Public TB KAP Questionnaire (CDC-TBKAPQ) developed by the Chinese Center for Disease Control and Prevention (*r* = 0.59*,* 0.60, *p* < 0.001). The receiver operating characteristics curve (ROC) analysis suggested a cut-off point of 47.5, with which the CS-TBKAPQ showed a sensitivity of 73.63% and a specificity of 80.51% in identifying students with low-level KAP. The positive and negative predictive values were 83.23% and 69.91%.

**Conclusions:**

The findings of this study demonstrate that the CS-TBKAPQ is a reliable and valid tool for measuring the KAP towards TB in college students.

## Background

Tuberculosis (TB) remains a major global health problem, especially in the Southeast Asia and Western Pacific regions [1]. China ranks third among the 22 high-burden countries and faces challenges in protecting high-risk, vulnerable and special populations from TB [1]. College students have become a high-risk group for TB [2]. Severe endocrine system fluctuations [3], high population density, close contact, and greater mobility in vocations are contributing factors towards TB infection and transmission in college students. According to the China Diseases Monitoring Information Report Management System, 37,040 students suffered from TB in 2013, which represents 4.10% of the country’s total TB patients. Among all students infected with TB, 85% were between 15 and 24 years old.

Interest in TB KAP research among college students has recently grown, especially using cross-sectional studies. TB is often neglected due to its atypical early symptoms [4]. Poor knowledge and attitudes cause delays in seeking cures and reporting to the medical staffs at schools, leading to a high prevalence of TB in colleges [5]. Limited physical exercise, poor nutrition and irregular routines aggravate TB infection [6].

The most common approach in KAP assessment is the questionnaire. There are several useful tools for measuring the KAP towards TB in cross-sectional surveys. Scholars have developed items through expert consultation [7]. They have also evaluated clarity and intelligibility by pre-testing [8, 9] and have tested internal consistency reliability [10]. However, a questionnaire based on the scale development steps has not yet been reported. There is no specific research on questionnaire development relevant to TB KAP for college students. The World Health Organization (WHO) published the Advocacy, Communication and Social Mobilization TB KAP Survey Questionnaire (WHO-TBKAPQ) [11]. The Public TB KAP Questionnaire (CDC-TBKAPQ), a widely used tool in China, was developed by the Chinese Center for Disease Control and Prevention (CDC) in 2006. These two generic questionnaires are widely used in public KAP surveys and focus on general knowledge, attitudes towards TB patients and health education, as well as the practices of initiative learning and knowledge dissemination. College campuses are high-density locations that create favourable conditions for TB transmission [12]. The crowded situation in both classrooms and dormitories increases the risk of transmission or an epidemic outbreak. Seeking medical services in a timely manner after disease onset, reporting the disease to the school staff, coordination of disease screening and preventive treatment will be a focus of the questionnaire [8]. Accordingly, it is important to develop a KAP questionnaire specifically for college students.

Based on “Advocacy, Communication and Social Mobilization for TB control-A Guide to Developing KAP Surveys” published by WHO [11], we developed the college students’ TB KAP questionnaire (CS-TBKAPQ) by following the steps of literature review, expert consultation, and pre-testing. We then assessed the reliability, validity, and diagnostic accuracy of the CS-TBKAPQ using factor analysis, correlation analysis, and diagnostic testing.

## Methods

### Participants

#### Participants and setting

Participants were recruited from universities in Xi’an city of Shaanxi Province between March and June of 2016. The inclusion criteria were (1) college students, (2) non-medicine majors, (3) healthy without TB, and (4) never suffered from TB.

### Sample size

#### Requirement of diagnostic test on sample size

The following is the formula to determine the sample size needed to employ a diagnostic test: $n={\left(\frac{z_{\alpha }}{\delta}\right)}^2\left(1-p\right)p$.

Based on the assumption that *p* = 0.75 (representing sensitivity or specificity), *δ* = 0.08 (representing permitted minimum error), *α* = 0.05 (representing I type error, *z* = 1.96 accordingly), and a 10% non-respondent rate in our estimate, a total sample size of 249 was necessary.

#### Requirement for a factor analysis of the sample size

The minimum sample size recommended is five participants per item. As the number of the items was intended to be 30, a minimum of 150 participants were needed [13]. The EFA sample and the CFA sample should be two independent samples, the sizes of which should not be fewer than 200 [14] and 300 participants, respectively. Given a 10% non-response rate, a total sample of 550 participants was needed. According to the requirements of both the diagnostic test and the factor analysis, the minimum sample size was set at 550 participants.

### Sample technique

Simple random sampling was adopted, in which six of the 63 universities in Xi’an city of Shaanxi Province were selected using a table of random numbers. Then, 100 college students were selected according to student ID number using a systematic sampling technique from each of the selected universities.

### Procedure

#### Phase 1: Framework for the development of the CS-TBKAPQ

The CS-TBKAPQ was developed with reference to the Theory of Knowledge, Attitude, Belief, and Practice (KABP or KAP). According to this theory, appropriate health care knowledge is the foundation for developing positive and correct beliefs and attitudes and therefore for improving health-related behaviours. On the basis of this theory, the framework of the CS-TBKAPQ was divided into three sections: knowledge, attitudes, and practices towards TB.

#### Phase 2: Item generation

The original items of the CS-TBKAPQ were generated from the results of literature reviews and individual interviews. The literature reviews covered domestic and foreign studies on KAP towards TB and questionnaire development. The research team conducted ten interviews in total. The personal in-depth semi-structured interviews were conducted on issues such as TB health education, TB discovery and screening, and questionnaire item development. Each interview lasted 20 to 30 min. The interviewees were one institute director, one division director and two other staff members of the TB Prevention Institute of Shaanxi Province, as well as two infectious disease administrators, two physicians and two radiologists from the university hospital.

### Phase 3: Item screening

#### Delphi technique

Two rounds of consultations were conducted by experts in related fields via email. The experts were asked to rate all items using a five-point Likert scale, with five being the most important and one being the least important (including “neutral”). The mean and standard deviation of the score of each item were calculated. The variation coefficient and item selection rate were chosen as item screening indices. The item selection rate was calculated by the proportion of items given a rating of 4 or 5 by all experts. The screening standard was “the item whose variation coefficient ≥ 0.20 or selection rate < 80% shall be deleted”.

### Phase 4: Pre-testing

To test the clarity and intelligibility of each item, the pre-final version of the CS-TBKAPQ was distributed to a sample of 40 college students.

### Measures

**Fig. 1:**
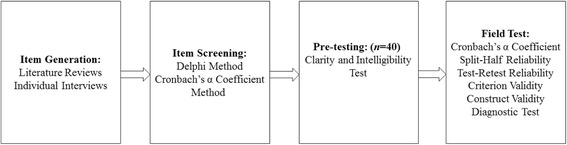
Flowchart of the study

The questionnaire was composed of four parts: demographic data, knowledge, attitudes, and practices towards TB. Each question of the knowledge section was rated in such a way that a score of one was given to correct responses and a score of zero was used for incorrect/don’t know responses. The score of multiple-choice questions was cumulative. A Likert scale was used to quantify the results of the other two parts. A five-point Likert scale was utilized from 1 (completely disagree) to 5 (completely agree) with a neutral midpoint for each item of the attitudes section. A four-point Likert scale was utilized from 1 (completely disagree or never) to 4 (completely agree or always) for each item of the practices section. Questions closely related to individual subjective feelings such as “first response after suffering TB”, “the reasons to refuse to participate in TB health education activity”, and “the reasons to refuse timely treatment after TB infection” were scored zero. The flowchart of the study is shown in Fig. 1.

### Data analysis

SPSS version 18.0 and Amos 17.0 were used for analysis. The questionnaires that were not filled out completely were regarded as invalid, and their data were discarded.

1. Descriptive statistics were used to summarize the demographic data.
2. Reliability of the CS-TBKAPQ was assessed by calculating Cronbach’s α coefficient, the split-half reliability coefficient and the test-retest reliability coefficient. Split-half reliability was measured by numbering KAP items of the questionnaire and dividing them into two parts containing odd and even numbers. The simple correlation coefficient of the scores of the two parts was then calculated. The test-retest correlations were determined by calculating the interclass correlation coefficient (ICC) of the scores of the two surveys conducted among the same group of students in a two-week interval. The statistically acceptable reliability coefficient should be >0.70 [15, 16].
3. Content validity was evaluated in the second round of expert consultation. The experts were asked to validate the relevance and feasibility of each item on a four-point Likert scale. The CVI was calculated by the proportion of items given a rating of 3 or 4 by all experts. The CVI should often be >0.70 [15, 17].
4. Construct validity of the CS-TBKAPQ was assessed by EFA. Factors were extracted by principal component factor analysis with a varimax orthogonal rotation method. Bartlett’s test of Sphericity and a Kaiser-Meyer-Olkin (KMO) test must be conducted to confirm the suitability of data [18]. Bartlett’s test of Sphericity with *p <* 0.05 and a KMO value of 0.60 were suitable when running the EFA [15]. There are two useful techniques in factor extraction: the Kaiser’s criterion for factors with an eigenvalue >1 and the Cattell’s scree test. Cattell recommends in the scree test that all factors above the elbow, or break in the plot, should be retained [19]. Factor loadings greater than 0.40 were considered significant.
5. Confirmatory factor analysis (CFA) was conducted to confirm the factorial structure of the CS-TBKAPQ identified in the EFA. As the scores did not follow a normal distribution, the generalized least squares (GLS) method was adopted to conduct parameter estimation [19]. Model fit indices, such as NC (normed Chi-square = *x* ^*2*^ */df*, *df* stands for ‘degrees of freedom’), GFI (goodness of fit index), AGFI (adjusted goodness of fit index), RMSEA (root mean square error of approximation) and RMR (root mean square residual) were used for model goodness-of-fit assessment. The model fit is acceptable if NC (*x* ^*2*^ */df*) < 3 (*p* > 0.05), GFI > 0.90, AGFI >0.90, RMSEA <0.08 and RMR < 0.05 [15].
6. Criterion validity was assessed by testing the correlations between the scores of the overall CS-TBKAPQ, the knowledge element, the attitudes element, the practices and the two other generic measures used in the public TB KAP survey: the CDC-TBKAPQ and the WHO-TBKAPQ. As the scores of the three questionnaires did not follow a normal distribution, the Spearman rank correlation coefficient was calculated.
7. Diagnostic accuracy assessment included two aspects: First, a comparative analysis was conducted on the sensitivity and specificity of the CS-TBKAPQ and the CDC-TBKAPQ. The CDC-TBKAPQ was a generic questionnaire and was a widely used tool in public KAP surveys in China. The theoretical assumption was that the specificity of the CS-TBKAPQ was higher than that of the CDC-TBKAPQ. Second, the diagnostic accuracy of the CS-TBKAPQ was evaluated using the calculated sensitivity, the specificity, the positive predictive value (PPV) and the negative predictive value (NPV).

### Reference standard selection

In this research, the WHO-TBKAPQ was selected as the reference standard of the diagnostic test. The WHO-TBKAPQ was developed by the WHO and can be considered the most widely accepted questionnaire among the existing tools in KAP assessment. Furthermore, the WHO-TBKAPQ has been used with modifications by some countries and has demonstrated good reliability, sensitivity and cultural acceptability. For instance, Daniel Tolossa applied the WHO-TBKAPQ to investigate community knowledge, attitudes, and practices towards TB in Ethiopia [4].

### Receiver operator characteristic (ROC) curve analysis

The participants who filled in each of the three questionnaires, namely, the WHO-TBKAPQ, CDC-TBKAPQ and CS-TBKAPQ, were divided into two groups based on the median of their scores. The participants who scored above the median were recognized to have high KAP levels, and the participants who scored below the median were recognized as having low KAP levels. Using the score of the WHO-TBKAPQ as criteria, the ROC curves of the scores of the CDC-TBKAPQ and the CS-TBKAPQ were drawn to determine the cut-off points of the two questionnaires.

### Calculation of diagnostic test index

The sensitivity of the diagnostic test indicates the percentage of college students who were correctly classified by the test as having low-level KAP. The PPV is the percentage of college students with a positive test outcome who actually have low-level KAP.

## Results

### Delphi technique

The 12 experts were from the TB prevention, clinical treatment, health education and college TB management fields. Their average age was 49.83 years (SD = 10.85), and the average length of service was 15.25 years (SD = 7.97). The experts with titles indicating senior professional posts accounted for 75%. Their academic backgrounds were closely related to the CS-TBKAPQ development. Following the first round, four items were deleted, as the selection rate was <80% or the variation coefficient was ≥0.2. The deleted items related to knowledge of TB infectivity, attitudes towards self-probability of TB infection, co-epidemics of TB and HIV, and attitudes towards TB patients. Two items related to the purpose of the purified protein derivative (PPD) test and the vaccine to prevent TB were added in the first round. In the second round, the two newly added items were deleted due to the selection rate of <80%. The initial questionnaire with twenty-four KAP items was developed through two rounds of the Delphi technique (Table 1).

**Table 1:** Results of the Delphi Technique

Round	Original items	Items unmodified	Items modified	Items deleted	Added items	Result
1	28	14	9	4	2	26
2	26	24	0	2	0	24

### Pre-testing

Forty new students of Xi’an Jiaotong University participated in the pre-testing and completed the questionnaire within 13 min. After revising the descriptions of some items according to the feedback, the pre-final questionnaire was determined. This questionnaire consisted of twenty-four KAP items.

### Sampling

A total of 710 college students participated in the field test through simple random sampling, which was more than the minimum required number of participants. The participants filled out the CS-TBKAPQ, the CDC-TBKAPQ and the WHO-TBKAPQ at the same time. Because fifteen participants did not complete the CS-TBKAPQ, the sample size of the valid CS-TBKAPQ was 695. The 695 data sets were used to assess reliability by calculating Cronbach’s α coefficient, the split-half reliability coefficient, and the test-retest reliability coefficient. The 695 data sets were also randomly divided into two groups. EFA was performed for the N1 dataset (*n* = 347), and CFA was performed for the N2 dataset (*n* = 348). Each participant was required to write in his/her student ID when filling out the questionnaire, and each questionnaire was given an ID from ‘001’ to ‘710’. Sixty questionnaires were randomly selected based on the questionnaire ID by systematic sampling. The questionnaire IDs were then matched with the student IDs to identify the participants. The sixty selected students were recruited in the retest study two weeks later, and all of the participants completed the questionnaires.

In the criterion validity assessment and the diagnostic test, the research team compared the results of the CS-TBKAPQ and the CDC-TBKAPQ using the WHO-TBKAPQ as the reference standard. The participant’s data were useful if all three questionnaires were completed. There were 69 participants who did not finish all three questionnaires. Thus, the sample size was 641 for the criterion validity assessment and the diagnostic test.

### Demographic characteristics

The minimum, maximum, mean and standard deviation of the CS-TBKAPQ scores (*n* = 695) were 62, 12, 46.54 and 8.57, respectively. We demonstrated the demographic characteristics of the samples of the EFA, CFA and diagnostic test in Table 2. In the EFA (N1 = 347) and CFA samples (N2 = 348), differences in demographic characteristics such as gender, age, nationality, residence, health education history and contact history with TB patients had no statistical significance.

**Table 2:** Demographic Characteristics of Participants

	EFA (N1 = 347)	CFA (N2 = 348)	*x* ^*2*^ */z*	*p*	Diagnostic test (*n* = 641)
**Gender**			0.53	0.47	
Male	152	162			297
Female	195	186			344
**Age (M ± Q)**	20 ± 3	20 ± 3	−1.78	0.08	20 ± 3
**Nationality**			0.04	0.85	
Han	331	333			611
Minority	16	15			30
**Residence**			1.05	0.31	
Urban	169	156			306
Rural	178	192			335
**Dormitory type**			2.04	0.56	
4-person	107	100			190
6-person	153	161			286
8-person	52	60			107
Other	35	27			58
**Monthly living expense**			2.48	0.65	
< 500 yuan	41	44			77
500–1000 yuan	140	143			255
1001–1500 yuan	117	102			207
1501–2000 yuan	33	43			74
> 2000 yuan	16	16			28
**Father’s education background**			4.63	0.20	
Primary School	20	28			44
Junior High School	100	118			194
Senior High School	139	118			241
Bachelor’s Degree	88	84			162
**Mother’s education background**			2.19	0.54	
Primary School	52	57			103
Junior High School	102	114			190
Senior High School	109	107			201
Bachelor’s Degree	84	70			147
**Past learning experience**			0.01	0.91	
Yes	162	164			298
No	185	184			343
**Past contact**			0.00	0.99	
Yes	60	60			111
No	287	288			530
Total	347	348			641

### Reliability

#### Cronbach’s α coefficient

The change analysis assesses the effect of deleting an item on the Cronbach’s α coefficient. After deleting the three items “what do you think of the prevalence of TB in the past and present period”, “what’s your response to your knowledge that your classmate suffers TB” and “behaviour of preventing TB”, the Cronbach’s α coefficient showed values of 0.83, 0.83 and 0.85, respectively, manifesting a relatively high increase compared with the overall value (0.82). Therefore, the three items were deleted. The final questionnaire was then developed. This questionnaire consisted of 31 items, including ten demographic characteristic items and twenty-one KAP items, eighteen of which were scoring items.

The Cronbach’s α coefficients were 0.86 for the total questionnaire, 0.42 for the knowledge element, 0.92 for the attitudes element, and 0.73 for the practices element. The split-half reliability coefficient was 0.78. Test-retest reliability estimated from ICC was 0.91 (95%CI: 0.781–0.995).

### Content validity

The CVI was 0.89, which achieved Lynn’s [20] criterion for content validity, indicating that the questionnaire can reflect the content of college students’ KAP towards TB.

### Exploratory factor analysis

**Fig. 2:**
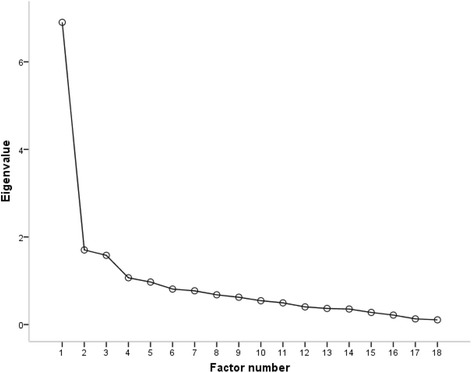
Cattell’s Scree Plot

The KMO value of Sample N1 (*n* = 347) was 0.91, and the result of Bartlett’s test of Sphericity was 3299.08 (*p <* 0.001), which indicated that the data were suitable for factor analysis. The results of the EFA revealed four eigenvalues greater than 1.0. An inspection of Cattell’s scree plot (Fig. 2) revealed a clear break after the fourth component. Therefore, four components were retained, which accounted for 62.52% of the variance. The factor loading with each item was above 0.4 without cross-loadings. According to the factor loading result and the item content, the first factor (F1) was named prevention attitude and behaviour, which involved nine items; the second factor (F2) was named active learning and treatment behaviour, which involved three items; the third factor (F3) was named disease knowledge, which involved four items; and the fourth factor (F4) was named treatment knowledge, which involved two items (Table 3).

**Table 3:** Factor Loading Results of the Exploratory Factor Analysis

Item		F1	F2	F3	F4
A5	College students should participate in a TB health education activity held by the school.	0.896	0.142	0.104	−0.013
A4	The school should hold a TB health education activity.	0.884	0.083	0.108	0.006
A8	After discovering an epidemic outbreak of TB, students should coordinate the field investigation of a disease control institution.	0.882	0.072	0.183	−0.004
A7	College students should coordinate TB screening held by the school.	0.866	0.132	0.152	−0.028
A3	College students should know relevant TB information.	0.831	0.043	0.150	0.025
A9	What is your attitude if the doctor suggests “preventative treatment” when a PPD test is discovered to be positive?	0.817	0.164	0.088	0.062
P6	You will actively accept a physical examination when your classmate suffers from TB.	0.748	0.112	0.051	0.015
P5	You remind your classmates to have a physical examination when you suffer from TB.	0.666	0.305	0.089	0.000
A2	College students with confirmed TB should tell a counsellor about the disease.	0.557	0.150	0.131	−0.071
P1	Will you actively increase your TB knowledge?	0.132	0.834	0.001	0.020
P2	Will you actively tell your family members or friends the TB knowledge you have gained?	0.230	0.803	0.119	0.001
P3	You will see a doctor in a timely fashion when you discover suspicious TB symptoms.	0.400	0.554	0.023	0.029
K3	Which of the following symptoms is a sign of TB?	0.121	0.045	0.778	0.084
K1	What is the transmission route of TB?	0.060	0.042	0.725	0.323
K2	What is a TB-vulnerable student?	0.233	−0.027	0.680	−0.035
K5	Free TB treatment institution.	0.096	0.106	0.463	−0.271
K4	Can TB be cured?	0.185	−0.138	0.066	0.804
K6	Free TB examination and treatment policy	−0.190	0.301	0.040	0.547
Eigenvalue		6.902	1.704	1.581	1.068
Contribution rate (%)		38.346	9.464	8.781	5.931
Cumulative contribution rate (%)		38.346	47.810	56.591	62.523

### Confirmatory factor analysis

**Fig. 3:**
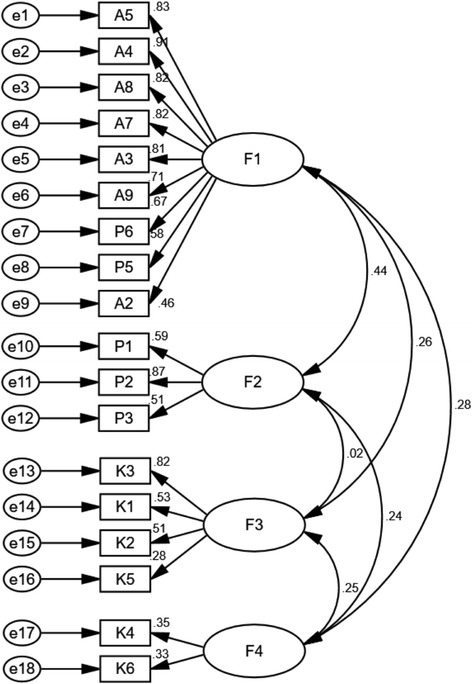
Path Diagram of the Confirmatory Factor Analysis

The CFA model of the CS-TBKAPQ was composed of four factors and 18 items, including F1 (items A5, A4, A8, A7, A3, A9, P5, P7, and A2), F2 (items P1, P2, and P3), F3 (items K4, K2, K3, and K6), and F4 (items K5 and K7). The parameter estimation results of GLS showed that no negative error variables appeared in the model and that all parameter estimations reached a significant level. Additionally, the estimation results did not contradict the model identification rule. The four-factor model failed to achieve exact fit (*x* ^*2*^ */ df* = 1.82, *p <* 0.001). An acceptable fit was indicated by GFI = 0.925, AGFI = 0.900, RMSEA = 0.049 and RMR = 0.068 [15]. All of the standardized factor loadings were statistically significant and greater than 0.40 (except items K4, K5 and K6). The parameter estimates of the CFA are shown in Fig. 3.

### Criterion validity

The CS-TBKAPQ showed significant positive correlation with the WHO-TBKAPQ and the CDC-TBKAPQ (*r* = 0.59*,* 0.60, respectively, *p* < 0.001). The knowledge items showed moderate correlation with the WHO-TBKAPQ and the CDC-TBKAPQ (*r* = 0.50*,*0.55, respectively, *p* < 0.001). The attitudes items showed moderate correlation with the WHO-TBKAPQ and the CDC-TBKAPQ (*r* = 0.47 and 0.46*,* respectively, *p* < 0.001). The practices items showed moderate correlation with the WHO-TBKAPQ and the CDC-TBKAPQ (*r* = 0.43 and 0.40, respectively, *p* < 0.001).

### Diagnostic test

**Fig. 4:**
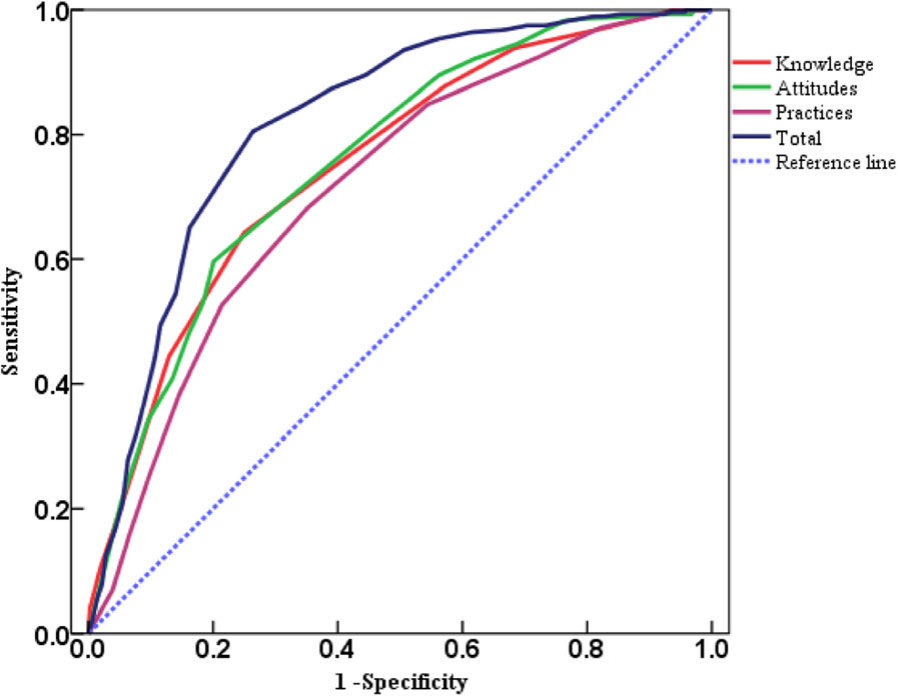
Roc Curve of the CS-TBKAPQ

Figure 4 presented the ROC curve computed for the scores of the CS-TBKAPQ and its three parts using the WHO-TBKAPQ score as criteria. The areas under the ROC curve for the CDC-TBKAPQ and the CS-TBKAPQ were 0.80 (95% CI: 0.767–0.836) and 0.82 (95% CI: 0.788–0.853), respectively. The best cut-off point of the CDC-TBKAPQ for screening purposes was 13.5, which resulted in a sensitivity of 71.70% and a specificity of 72.56%. The best cut-off point of the CS-TBKAPQ was 47.5, which resulted in a sensitivity of 73.63%, a specificity of 80.51%, a PPV of 83.23%, and an NPV of 69.91%, suggesting a good yield for discriminating between the students with low KAP levels from those with higher levels. In total, of the 641 college students surveyed, 322 (50.20%) had low KAP levels, and 319 (49.80%) had relatively high KAP levels. According to the ROC curve result, the best cut-off points for the knowledge, attitudes and practices elements of the CS-TBKAPQ were 5.5, 28.50 and 13.50, respectively. The analysis result is shown in Table 4.

**Table 4:** Diagnostic Test Results of the CS-TBKAPQ and CDC-TBKAPQ

	Sensitivity (%)	Specificity (%)	Diagnosis result	Reference standard		Total	PPV (%)	NPV (%)
				Low	High			
CS-TBKAPQ	73.63	80.51	Low	268	54	322	83.23	69.91
			High	96	223	319		
			Total	364	277	641		
CDC-TBKAPQ	71.70	72.56	Low	261	76	337	77.45	66.12
			High	103	201	304		
			Total	364	277	641		

## Discussion

To our knowledge, research focusing on questionnaire development of college students’ KAP towards TB is limited. Additionally, few tools have been developed for this specific purpose following the scale development steps. In this study, we produced a 31-item CS-TBKAPQ and evaluated its reliability, validity and diagnostic accuracy. The CS-TBKAPQ is more consistent with real-world conditions than the WHO-TBKAPQ in assessing TB prevention and control among college students in China. The CS-TBKAPQ performs better than the CDC-TBKAPQ in diagnosing low-level KAP students.

Cronbach’s α coefficient, the split-half reliability coefficient and the test-retest reliability coefficient were above 0.7, showing acceptable internal consistency and reliability [15, 16, 20]. The construct validity analysis showed that the four factors extracted in EFA explained 62.52% of the total variance, which was higher than the criterion of 50%. All factor loadings were above 0.4, revealing close relations between factors and items [19]. The four-factor model of CFA failed to perfectly fit the collected data as demonstrated by the highly significant Chi-square test for exact fit; however, this test is known to be overly sensitive to sample size [16]. Approximate fit indices were proposed to overcome this difficulty. The RMR value of 0.068 was deemed acceptable, and the other model fit indices reached the ideal standard, therefore demonstrating highly acceptable model fit with the observed data. CFA and EFA verified that the CS-TBKAPQ had reasonable construct validity.

The criterion validity analysis demonstrated that the CS-TBKAPQ had moderate correlations with the WHO-TBKAPQ and the CDC-TBKAPQ [20]. Some items of the three questionnaires overlapped in topics, such as the core knowledge of TB, the attitudes towards health education, the practices of active learning and knowledge dissemination. Thus, several new items that reflected characteristics of college TB control were added in the CS-TBKAPQ. The newly added items included the knowledge item “TB susceptible population among students”; the attitudes items regarding informing the university when you suffer TB, coordinate TB screening and a field investigation by the CDC in an epidemic outbreak; as well as the practices items “remind surrounding students to conduct physical examination, and seek help from doctors if your classmates suffer TB” and “timely treatment after discovering suspicious symptoms”. These items embodied the differences between the CS-TBKAPQ and the two general questionnaires.

The diagnostic test showed that the specificity (80.51%) of the CS-TBKAPQ was higher than that of the CDC-TBKAPQ (72.56%), which verified the theoretical assumption that “the specificity of the questionnaire specially developed for college students shall be higher than the CDC-TBKAPQ developed for the public”. The diagnostic test also proved the necessity of developing the CS-TBKAPQ. The sensitivity and specificity of the CS-TBKAPQ were 73.63% and 80.51%, respectively, suggesting that the CS-TBKAPQ is a valuable tool for identifying students with low KAP levels [21, 22]. We found that 50.20% of 641 college students had low KAP levels and that 49.80% of them had relatively high KAP levels, indicating a high proportion of students with low KAP levels and demonstrating a lack of sufficient knowledge, positive attitudes and sound practices regarding TB. This conclusion has good consistency with the findings by Faustine Nkulu [23] that “the proportions with good knowledge and positive attitudes of 16-24 years old students were 11.1% and 44.4%, the proportions with good knowledge and positive attitudes of students having more than 12 years of education were 23.2% and 55.8%”. This finding was also consistent with Milos Smolovic’s [9] statement that students not having sufficient knowledge about the cause and transmission mode of TB should be prioritized in TB health education.

The CS-TBKAPQ also indicated potential targeted interventions to improve KAP levels in its assessment of the cut-off points for knowledge, attitudes and practices. For instance, it is important for students with insufficient knowledge to participate in TB-related health education curricula [8], and those with poor attitudes should be guided through peer education to strengthen TB knowledge internalization [4]. In addition, students without sufficient practice should be encouraged to participate in TB prevention-themed activities and to improve their knowledge and attitudes towards health practices [8, 12].

TB prevention and control among college students has become an important public health issue in China. Evidence has shown that health education on KAP can help reduce the incidence and burden of TB. It can also help in early detection, isolation and treatment to prevent outbreaks. The study provides a simple, practical tool, the CS-TBKAPQ, to evaluate the KAP level among college students. This tool can be used in baseline data collection for targeted health education and intervention.

There are some limitations to this research. Only universities in Shaanxi Province were selected for the field test. The samples can be expanded to the other regions of China in further studies. In the knowledge section, Cronbach’s α coefficient was 0.42, which failed to meet the requirement of no less than 0.70, which may be due to the knowledge items covering TB symptoms, transmission and treatment policies. The overall Cronbach’s α coefficient was 0.86. Therefore, the reliability of the internal consistency of the questionnaire was considered adequate.

## Conclusions

The CS-TBKAPQ is a theory-based tool that was developed following the scale development steps. The study demonstrated that the CS-TBKAPQ has good internal consistency, test-retest reliability, content validity, construct validity, and criterion validity. As a specific TB KAP measure for college students, the CS-TBKAPQ is more sensitive and specific than the generic measure based on the diagnostic test results. This questionnaire can be used to investigate the TB KAP level of college students in China and to provide baseline data for health education or the evaluation of health education [24]. In addition, it can also be used to analyse the association between KAP and socio-economic factors [5].

## Abbreviations

AIID: Cronbach’s alpha coefficient if item deleted
CFA: Confirmatory factor analysis
CVI: Content validity index
EFA: Exploratory factor analysis
GLS: Generalized Least Squares method
ICC: Interclass correlation coefficient
KAP: Knowledge, attitudes, and practices
NPV: Negative predictive value
PPV: Positive predictive value
ROC: Receiver operator characteristic curve
TB: Tuberculosis
